# A longitudinal study of the COVID-19 pandemic impact on mental health in ophthalmic personnel and students

**DOI:** 10.1371/journal.pone.0300144

**Published:** 2024-03-13

**Authors:** Yi Pang, Connor Robbs, Jingyun Wang

**Affiliations:** 1 Illinois College of Optometry, Chicago, IL, United States of America; 2 State University of New York College of Optometry, New York, NY, United States of America; Uniformed Services University: Uniformed Services University of the Health Sciences, UNITED STATES

## Abstract

**Background:**

Our previous study revealed that the COVID-19 pandemic posed mental health challenges to eye care professionals and students. The intent of this study was to identify the longitudinal impact of the COVID-19 pandemic on mental health among ophthalmic personnel and students. Additionally, the potential risk factors for mental health problems were investigated.

**Methods:**

A two-phase survey among eye care professionals and students in the USA and Canada was conducted. Phase 1 was administrated from June 23 to July 8, 2020, and has been published; Phase 2 was conducted from January 21 to February 2, 2021. A total of 824 eye care professionals and students participated in Phase 2, with a response rate of 44.1%. Symptoms of depression, anxiety, and stress were measured and calculated.

**Results:**

Compared with Phase 1, stress scores in Phase 2 were significantly reduced (*P*<0.001) although they were still higher than the pre-COVID level (*P* < .001). Scores for depression (*P* = 0.20) and anxiety (*P* = 0.40) showed no change, comparing Phase 2 to Phase 1. Reduction of stress scores significantly differed among occupations (*P* = 0.005); students had less reduction of stress than other eye care professionals (all *P*<0.05). Vaccination status was significantly associated with a reduction in stress scores (*P* = 0.04).

**Conclusions:**

With the continuing COVID pandemic, although stress level was reduced at 7-month follow-up, the mental health of eye care professionals was still impacted. In this population, COVID-19 vaccination was associated with fewer stress symptoms. These results indicate that the COVID-19 pandemic continues impacting mental health among eye care professionals, especially students. These study results warrant future interventions for eye care personnel and students to prevent or treat mental health disorders.

## Introduction

Mental health problems during the COVID-19 pandemic have been extensively studied [1–11]. Numerous studies have reported the burden of mental health problems among the general population as well as among healthcare providers during the pandemic [12–15]. Previously, we reported a high prevalence of mental health problems and revealed mental health disparities among eye care professionals [16]. Our previous findings suggest that it is crucial to detect mental health problems more effectively and develop interventions to address this important public health issue. In addition, COVID-19 dramatically impacted academic eye care including didactic and clinical teaching, patient care, and both clinical and basic research [17], which could further challenge optometry students and impact their mental health.

Although there are many studies to investigate mental health of health care professionals, there are very few papers reporting the long-term impact of COVID-19 on eye care professionals. The aims of this study were 1) to determine whether there was a change in mental health problems including depression, anxiety, and stress in eye care professionals, staff, and optometry students over 7 months during the COVID-19 pandemic; 2) to evaluate whether the COVID-19 vaccine status of individuals had an impact on their mental health status; 3) to study the following potential predictors on their mental health status: participants’ sex, age range, race, ethnicity, occupation, and childcare responsibilities.

## Methods

The study protocol and consent forms were approved by the Institutional Review Board of the Illinois College of Optometry (Chicago, IL) with Institutional Review Board number 19032. Adherence to the Health Insurance Portability and Accountability Act (HIPAA) was maintained.

This was a longitudinal, survey-based study. The Phase 1 survey was administered from June 23 to July 8, 2020, and has been published [16]. From January 21 to February 2, 2021, the Phase 2 survey (Table 1) was sent to the individuals who participated in the first survey and voluntarily provided their email address (n = 1869). Multiple email reminders were sent to increase the response rate. A total of 824 individuals responded to the Phase 2 survey with a response rate of 44.1%. Twelve participants were excluded due to missing critical data such as Phase 1 stress score, depression score, or Phase 2 vaccine status data. Therefore, data from 812 participants were analyzed.

**Table 1:** Phase 2 survey questions.

1. How do you define your gender? ☐ Male ☐ Female ☐ Non-Binary☐ Trans Male ☐ Trans Female ☐ Trans Non-Binary ☐ Identity Not Listed ☐ Decline to specify 2. How do you define your race? ☐ White/ Caucasian ☐ Black/ African American ☐ Asian ☐ American Indian/Alaska Native ☐ Native Hawaiian or Other Pacific Islander ☐ More than one race ☐ Decline to specify 3. How do you define your ethnicity? ☐ Hispanic/Latino ☐ Non-Hispanic/Latino ☐ Decline to specify 4. How do you define your stress level over the last 2 weeks in scale 1 to 5? (1 means no stress and 5 is highest in stress level)? ☐ 1 ☐ 2 ☐ 3 ☐ 4 ☐ 5 5. Do you have childcare responsibilities while working in the last 2 weeks? ☐ No, no childcare responsibilities ☐ Yes, <25% of childcare work is my responsibility ☐ Yes, 25–50% of childcare work is my responsibility ☐ Yes, 50–75% of childcare work is my responsibility ☐ Yes, >75% of childcare work is my responsibility Over the last 2 weeks, how often have you been bothered by any of the following problems listed from questions 6 to 9 6. Little interest or pleasure in doing things ☐ Not at all ☐ Several days ☐ More than half the days ☐ Nearly every day 7. Feeling down, depressed, or hopeless ☐ Not at all ☐ Several days ☐ More than half the days ☐ Nearly every day 8. Feeling nervous, anxious, or on edge? ☐ Not at all ☐ Several days ☐ More than half the days ☐ Nearly every day 9. Not being able to stop or control worrying ☐ Not at all ☐ Several days ☐ More than half the days ☐ Nearly every day 10. How do you describe your current job/position? ☐ Ophthalmologist in private practice, group practice, or hospital ☐ Ophthalmologist in academic setting ☐ Optometrist in private, group, or corporate practice ☐ Optometry student (interact with patients) ☐ Optometry student (no interaction with patients) ☐ Staff in clinic (interact with patients) ☐ Staff not in clinic (no interaction with patients) ☐ Faculty in clinic (interaction with patients) ☐ Faculty not in clinic (no interaction with patients) 11. What is your age? ☐ 20–29 ☐ 30–39 ☐ 40–49 ☐ 50–59 ☐ 60–69 ☐ 70–79 ☐ 80+ 12. Which state have you lived in the last two weeks? 13. How many days a week have you been interacting with patients during the last two weeks? ☐ No interact with patients ☐ 1 day ☐ 2 day ☐ 3 day ☐ 4 day ☐ 5 day or more 14. In the last 2 weeks, how often have you felt that you were unable to control the important things in your life? ☐ Never ☐ Almost never ☐ Sometimes ☐ Fairly often ☐ Very often 15. In the last 2 weeks, how often have you felt confident about your ability to handle your personal problems? ☐ Never ☐ Almost never ☐ Sometimes ☐ Fairly often ☐ Very often 16. In the last 2 weeks, how often have you felt that things were going your way? ☐ Never ☐ Almost never ☐ Sometimes ☐ Fairly often ☐ Very often 17. In the last 2 weeks, how often have you felt difficulties were piling up so high that you could not overcome them? ☐ Never ☐ Almost never ☐ Sometimes ☐ Fairly often ☐ Very often 18. How would you describe the impact of COVID 19 on your mental health? ☐ Overall, COVID 19 has a **negative** impact on my mental health ☐ Overall, COVID 19 has a **positive** impact on my mental health ☐ Overall, COVID 19 has no impact on my mental health 19. Have you received the first or second dose of any COVID-19 vaccine or are scheduled to receive a vaccine currently? a. Yes, I received 1st or 2nd dose of vaccine already. b. Yes, I have vaccine scheduled, but not received any doses yet. c. No, I have not received any dose of vaccine and have not gotten it scheduled.

### Predictor measures

Demographic information and information about potential predictors were collected, including sex, age range, race, ethnicity, vaccine status, occupation, and childcare responsibilities. Participants’ names were not collected. The survey ID (email) was utilized to assign each participant number. No time limit was given to participants to finish the survey. Participants were allowed to review the previous questions during the survey. We did not use the IP addresses of the participants’ computers to track survey entry. The order of survey questions was altered to reduce answering order biases.

### Mental health measures

Depression, anxiety, and psychological stress were assessed using validated questionnaires [18–20]. The questionnaire measurements and score calculations were described in our previous publication^1^. Probable cases of depression and anxiety were calculated based on the clinical threshold from the depression and anxiety scales (greater than or equal to 3 points on a 0–6-point scale), respectively. The threshold for detecting a probable case of depression and anxiety in each scale was a score greater than or equal to 3 [18].

### Sample size calculation

If the true difference in the mean stress score is 0.33 with a standard deviation of 1.227 in 812 participants (12 participants were excluded because of missing data), we would be able to reject the null hypothesis that this response difference is zero with probability (power) 1.000. The Type I error probability associated with this test of the null hypothesis is 0.05.

### Statistical analysis

All data were analyzed using the R 3.5.0 Statistics software (R Core Team. URL http://www.R-project.org/). Descriptive statistics were applied including mean and standard deviation of depression, stress, and anxiety scores. Shapiro-Wilk’s test was used to determine normality; we found the differences in these scores were not normally distributed and thus, we used nonparametric tests to analyze data. The differences in scores of stress, depression, and anxiety between Phase 1 and Phase 2 were calculated and further analyzed using the paired Wilcoxon test.

For the variables that significantly differed from the Phase 1 survey, we performed subgroup analyses stratified by gender, race, ethnicity, age, vaccine status, occupation, and childcare responsibility. The Kruskal-Wallis test was used to determine differences among the subgroups. When the Kruskal-Wallis test revealed a difference among the groups, post-hoc pairwise comparisons were performed using the Wilcoxon rank sum test. A P < 0.05 indicates statistical significance.

## Results

The demographic characteristics of the participants are listed in Table 2.

**Table 2:** Demographic characteristics of the participants (n = 812).

Characteristic		n (%)	
**Gender**			
	Female	599 (73.1%)	
	Male	204 (25.0%)	
	Non-binary/Trans non-binary/decline to specify	9 (0.2%)	
**Race**			
	White	510 (62.8%)	
	Black	30 (3.7%)	
	Asian	212 (26.1%)	
	American Indian/Alaska native	1 (0.1%)	
	Native Hawaiian or Other Pacific Islander	6 (0.7%)	
	More than one race	20 (2.5%)	
	Decline to specify	33(4.0%)	
**Ethnicity**			
	Hispanic/Latino	50 (6.2%)	
	Non-Hispanic/Latino	724 (89.1%)	
	Decline to specify	38 (4.7%)	
**Age (years)**		
	20–29	372 (45.8%)	
	30–39	187 (23.0%)	
	40–49	108 (13.3%)	
	50–59	72 (8.9%)	
	60–69	60 (7.4%)	
	70–79	7 (0.9%)	
	80+	6 (0.7%)	
**Vaccine status in Phase 2**			
At least 1 of 2 vaccine doses administered/scheduled		557 (68.6%)	
No vaccine scheduled		255 (31.4%)	
**Career distribution**			
Ophthalmologist		46 (5.7%)	
Optometrist		359 (44.2%)	
Optometry student		342 (42.1%)	
Eye care staff or non-clinical faculty		65 (8.0%)	
**Childcare responsibilities**			
	No childcare responsibilities	630 (77.6%)	
	Yes, <25% of childcare responsibility	32 (3.9%)	
	Yes, 25–50% of childcare responsibility	51 (6.3%)	
	Yes, 50–75% of childcare responsibility	46 (5.7%)	
	Yes, >75% of childcare responsibility	49(6.0%)	
	Missing data	4 (0.5%)	

### Scores on depression, anxiety, and psychological stress scale in Phase 2

In the Phase 2 survey, there were a total of 19 questions including 18 questions identical to the first survey and one additional question related to vaccination (Table 1). The depression and anxiety screening questions showed 37.6% of all participants (n = 812) having probable cases of either depression, anxiety, or both. This includes 35.1% of participants scoring higher than the threshold as probable cases of anxiety (score ≥ 3 on the 6-point scale), 18.2% scoring above the threshold as probable cases of depression (score ≥3 on the 6-point scale), and 15.8% scoring above the threshold for both.

### Overall stress, anxiety, and depression level in Phase 2 compared with Phase 1

**Fig 1:**
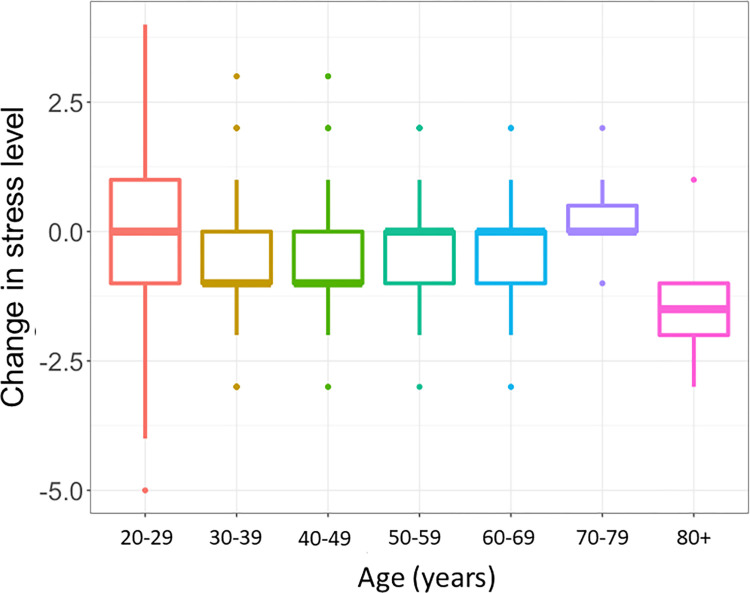
Change in stress scores according to age group (n = 812).

**Fig 1** summarizes the change in stress, depression, and anxiety from Phase 1 to Phase 2. Phase 2 stress scores were significantly lower than Phase 1 scores (3.21 ± 1.10 for Phase 2 and (3.54 ± 1.12 for Phase 1, *W* = 3e+05, *P* < .001). The depression scores did not change significantly between Phase 2 (1.38 ± 1.50) and Phase 1 (1.49 ±1.56) (*W* = 5e+05, *P* = .20). The anxiety level also did not change between Phase 2 (2.17 ± 1.75) and Phase 1 (2.24 ±1.78) (*W* = 3e+05, *P* = .40). Thus, only the stress scores were reduced at Phase 2 and the depression and anxiety scores were still high. We further studied potential key factors related to the significant change in stress scores.

**Fig 2:**
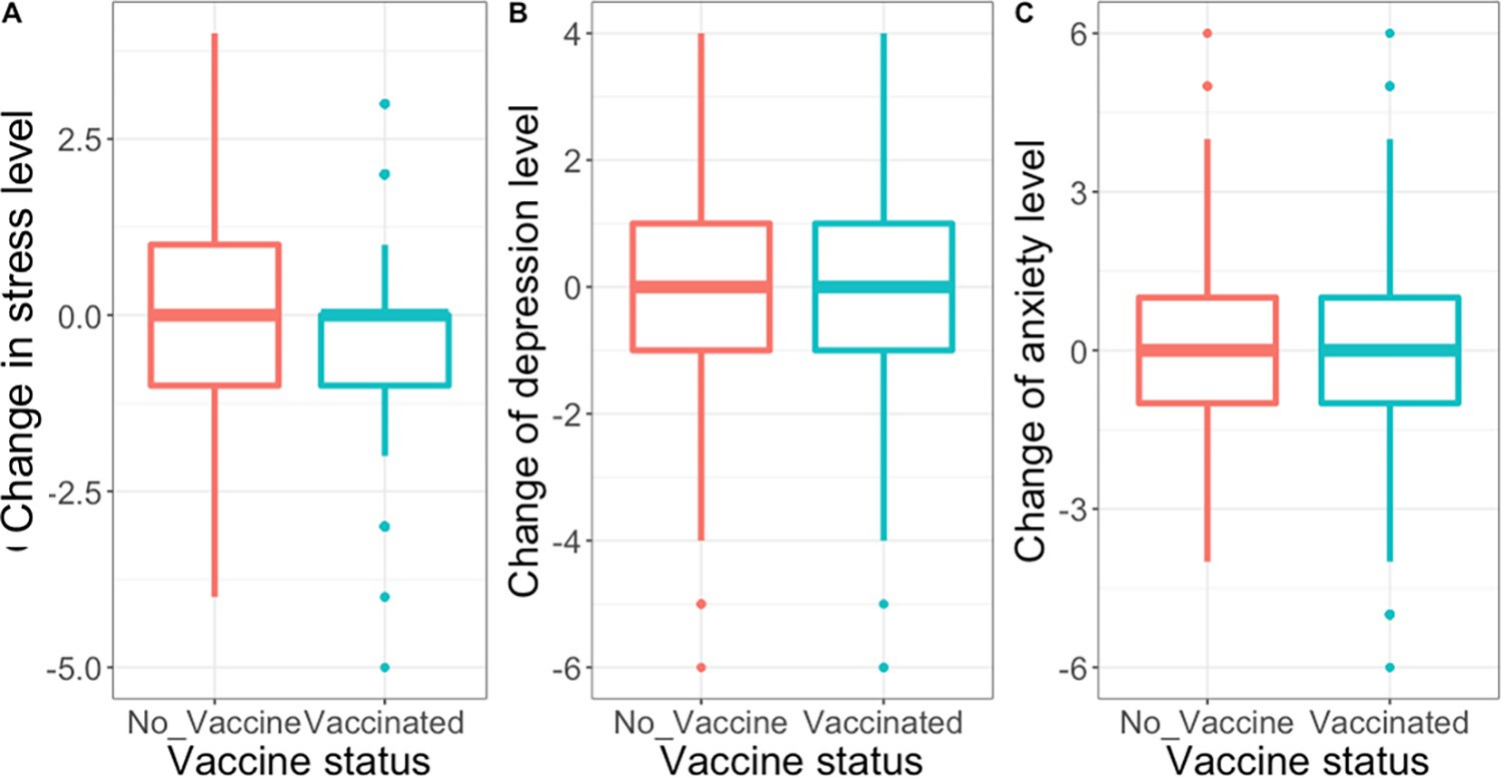
Changes in stress, depression, and anxiety scores according to vaccine status from Phase 1 (n = 812).

**Fig 3:**
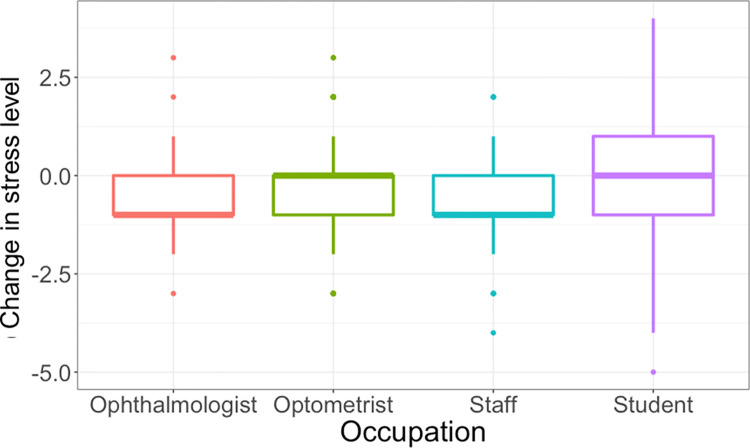
Change in stress scores according to occupation (n = 812).

- **Gender, race, and ethnicity:** The change in stress level between Phases 1 and 2 was not associated with gender (*χ*^2^ = 0.90, *P* = .60), race (*χ*^2^ = 8.00, *P* = .30), or ethnicity (*χ*^2^ = 4.00, *P* = .10).
- **Age Impact (Fig 1):** Age was significantly associated with changes in stress level between Phase 1 and Phase 2 (*χ*^2^ = 20.00, *P* = .006). Pairwise Wilcoxon tests showed the major difference was between the age-subgroup (20–29 YR) and age-subgroup (30–39 YR) (*P* = .03), indicating the change in stress level was less in the youngest group aged from 20 to 29 years when compared with other age subgroups.
- **Vaccine Impact (Fig 2):** By Phase 2, the stress level was significantly reduced by -0.40 ± 1.17 for those vaccinated or vaccine-scheduled compared to -0.20 ± 1.33 for the unvaccinated subgroup (*χ*^2^ = 4.00, *P* = .04). There was no statistically significant difference in change in depression (*χ*^2^ = 2.00, *P* = .20) or anxiety (*χ*^2^ = 0.20, *P* = .70) between vaccinated and unvaccinated individuals.
- **Occupation impact (Fig 3):** For the Phase 2 survey, the mean stress level was 3.02 ± 0.93 for ophthalmologists, 2.99 ± 1.08 for optometrists, 3.55 ± 1.04 for students, and 2.83 ± 1.17 for staff (*χ*^2^ = 60.00, *P*< .001). Pairwise Wilcoxon tests showed that students had significantly higher stress score compared with ophthalmologists (*P* = .001), optometrists (*P*< .001), and staff (*P*< .001). Fig 3 shows the mean change in stress level between Phase 1 and Phase 2 among occupations. The change in stress scores was statistically significantly associated with type of occupation (χ^2^ = 10.00, *P* = .005). Pairwise Wilcoxon tests showed significant differences between the students and optometrists (*P* = .03), and marginal difference between students and ophthalmologists (*P* = .05) as well as students and staff (*P* = .05).
- **Childcare responsibility:** There was no significant difference in the change in stress level related to childcare responsibility (*χ*^2^ = 4.00, *P* = .60).

## Discussion

To the best of our knowledge, this is the first longitudinal study to assess mental health status in eye care professionals in the United States during the COVID-19 pandemic. We found a high prevalence (37.6%) of probable depression, anxiety, or both among eye care professionals. The follow-up survey showed that stress was reduced significantly, especially for those vaccinated or scheduled to be vaccinated. Unfortunately, stress scores in vaccinated students were still high, which indicates that more attention and care should be given to students’ mental health. Interestingly, both depression and anxiety scores remained the same in the follow-up survey. Furthermore, vaccination status was not significantly associated with depression and anxiety scores. More research is warranted to determine the associated factors with mental health problems relative to eye care professionals.

Our previous study revealed that being Black, Asian, female, or young was associated with more mental health problems during the COVID-19 pandemic. Interestingly, having greater proportions of childcare responsibilities at home or more frequent interactions with patients appeared to be protective against depression [16]. This study focused on the changes in mental health over 7 months between Phase 1 and Phase 2. The stress level was significantly reduced at the follow-up; however, both depression and anxiety remained at a high level.

In Phase 2, our finding that students and the youngest eye care professionals showed less reduction of stress levels than other occupations and age subgroups is consistent with recent publications [21–26]. Younger physicians or adults reported higher mental stress [21, 22]. The psychosocial effects of COVID-19 disproportionately affect young people [23]. Du et al. also reported that students had higher stress levels during the COVID-19 pandemic [26]. A study conducted at Texas A&M University showed that 71.26% of 2031 total student participants reported that their stress/anxiety levels increased during the pandemic [27], which is consistent with our study findings. Our longitudinal study demonstrated that students and younger professionals are vulnerable to mental stress, consistent with our previous findings [16].

Although there was no association between vaccination status and anxiety or depression scores, we found that vaccination status was associated with stress level. According to a study on mental health in nurses, vaccination reduced anxiety and depression in an early-vaccination cohort [28, 29]. On the other hand, probably due to apprehension about vaccine efficacy, preliminary trials, and associated side effects, individuals might experience more stress or anxiety when making the vaccination decision or not [30, 31]. Our results are consistent with the findings that mental health issues related to COVID still require attention even with the availability of vaccination [32].

Efforts have been made to manage mental health problems in health care workers during the COVID-19 pandemic [33–38]. However, we found that 37.6% of eye care professionals still demonstrate potential clinical depression and anxiety, which indicates that eye care practitioners need further mental health support.

This study has several limitations. First, our study might underestimate the influence of the COVID-19 pandemic on mental health because the study was conducted before new variants such as delta and omicron emerged. New variants brought uncertainty and doubts about the vaccine [39], which might influence long-term mental health but is beyond the scope of this study. Our follow-up was limited to seven months. Second, this study enrolled a convenient sample of individuals based on professional connections, which may not represent the general eye care professional. The assessment of the vaccination status and stress is also subject to selection bias because the exposure was not randomly assigned. Our sample was 73.1% female, which may reflect a bias towards women. Female health care providers were reported to have a higher likelihood than male providers to experience mental health problems during the COVID-19 pandemic [40]. According to a narrative review on women’s mental health and COVID-19 [25], women demonstrate more risk factors known to increase during a pandemic [41–43]. It is challenging to achieve a high response proportion in external surveys, which have an average response proportion of about 10–15% [44]. Our Phase 2 study had a 44.1% response proportion, which is higher than the reported average proportion. Finally, both medical doctors (21.0%) [45] and optometrists (18.8%) [46] have a higher percentage of Asian than the general population (6.3%) [47], more Asians participated in our study (26.1%) than the general population.

### Conclusion

Stress level was significantly reduced in eye care personnel and students after 7 months, however not for depression and anxiety. Vaccinated participants reported a lower stress level compared to unvaccinated participants. These results indicate that the COVID-19 pandemic can continue impacting mental health among eye care professionals, especially students, for extended periods after the initial pandemic onset. This study suggests that additional research is warranted to better understand the impact of the COVID-19 pandemic relative to mental health so that future preventions and treatments can be implemented.

## Abbreviations

GAD-2: 2-item Generalized Anxiety Disorder scale (measuring anxiety disorder)
HIPPA: Health Insurance Portability and Accountability Act
IRB: Institutional Review Board
NPR: National Public Radio
OR: Odds Ratio
PHQ-2: 2-item Patient Health Questionnaire (measuring depression disorder)
PSS-4: 4-item Perceived Stress Scale
U.S.: United States of America
